# Microwave irradiation directly excites semiconductor catalyst to produce electric current or electron-holes pairs

**DOI:** 10.1038/s41598-019-41002-w

**Published:** 2019-04-02

**Authors:** Jicheng Zhou, Zhimin You, Wentao Xu, Zhiming Su, Yin Qiu, Lingfei Gao, Cheng Yin, Lixin Lan

**Affiliations:** Key Laboratory of Green Catalysis and Chemical Reaction Engineering of Hunan Province, School of Chemical Engineering, Xiangtan University, Xiangtan, 411105 Hunan Province PR China

## Abstract

Generally, photon of Microwave (MW) electromagnetic waves have long been thought to be lower energy, which could not excite metals or semiconductor materials to generate electric current and electron-holes pairs (e^−^_cb_ + h^+^_vb_). In this paper, we report an unexpected, Microwave “photoelectric effect”, when MW irradiates on the semiconductor materials, leading to generate electric current and electron-holes pairs (e^−^_cb_ + h^+^_vb_), on the semiconductor materials and on the MW catalyst. Further, we show that the action mechanism of Microwave “photoelectric effect” made water adsorbing on the surface of Microwave catalyst transform into hydroxyl radical (∙OH). Thus, this study has revealed the principle of generation Microwave “photoelectric effect” under MW irradiation, and the mechanism of MW catalytic oxidation degradation of organic in the wastewater and the mechanism of MW reduction method for preparation of nano-particle metal supported catalysts. Our findings challenge the classic view of MW irradiation only as heating method, which cannot excite to produce electric current and electron-holes pairs (e^−^_cb_ + h^+^_vb_). Our findings will open new field to use MW technology for MW catalytic oxidation degradation of organics in the wastewater, and for MW reduction method of metal supported catalysts preparation.

## Introduction

Microwave (MW) refers to the electromagnetic waves of 300 MHz-300 GHz, wavelength in meters (excluding 1 m) to 1 millimeter. Microwave technology has already been applied widely in many fields^1–3^, the effects of microwave accelerating chemical reaction were studied^1–14^. The application of microwave technology in the wastewater treatment is a new development^15,16^. The microwave plays the role of inducement, enhancement, and assistance degradation in the microwave induced catalytic degradation (MICD)^17^,the microwave-enhanced catalytic degradation (MECD)^18–20^, and the microwave assisted catalytic degradation method (MACD)^21–24^, Zhang *et al*.^24^. reported under MW irradiation to form powerful oxidizing •OH (and other ROS), which can non-selectively attack the organic contaminants, and possible principle on the degradation of parathion in A-TiO_2_/AC/MW and R-TiO_2_/AC/MW is that TiO_2_ can be excited by hot-spots to generate electron-hole pairs, and then, to form hydroxyl radicals (•OH) in aqueous solution. But to date it is still not clear that the nature of MW interact with MW catalyst for the wastewater treatment. El-Shall *et al*.^25^ has reported Microwave synthesis of supported Au and Pd nanoparticle catalysts. However, it is not well understand the mechanism of this method^26^. Herein, we found that microwave “photoelectric effect” exists when MW irradiates on the semiconductors and microwave “photoelectric effect” made water on the surface of Microwave catalyst to transform into hydroxyl radical (∙OH).Our finding revealed the mechanism of microwave catalytic oxidation reaction for degradation of organics and reduction of metal ion into metal on the oxide support under MW irradiation.

## Results and Discussion

**Figure 1:**
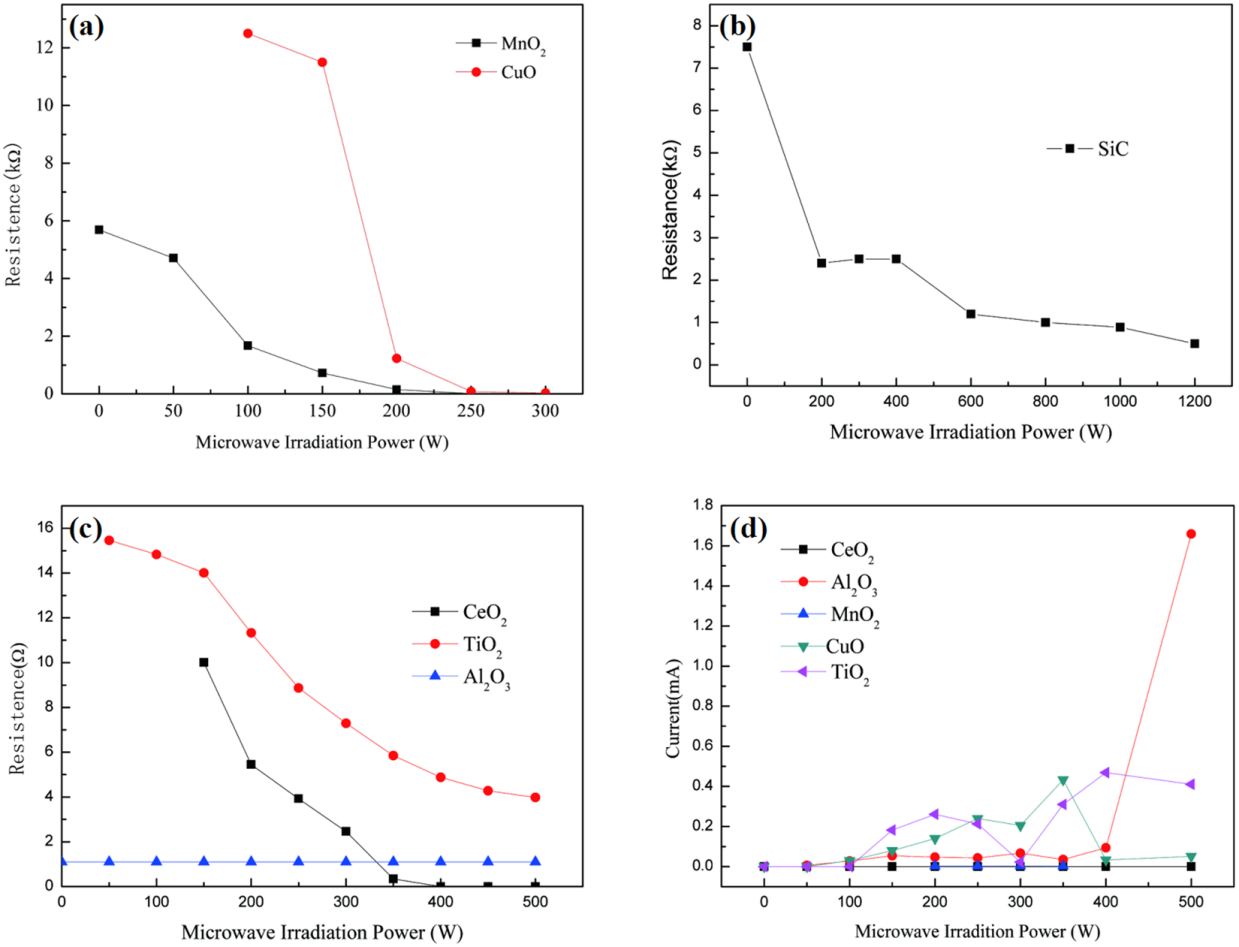
The change of conductivity with MW irradiation input power (P_MW_)(**a**–**c**) and the effect of P_MW_ on produced electric current (**d**).

**Figure 2:**
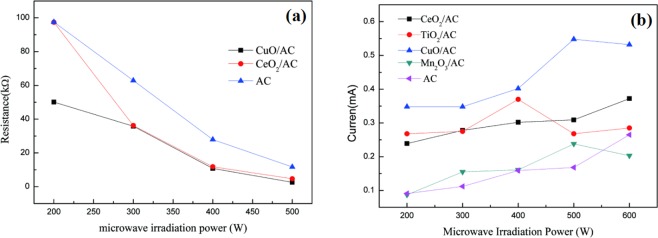
The change of conductivity with P_MW_(**a**) and the change of conductivity with P_MW_(**b**).

MW irradiation on semiconductor experiments were conducted using semiconductor TiO_2_,CuO,CeO_2_ and Mn_2_O_3_ as acceptor of MW photon, our observation is that the electrical conductivity of semiconductor CuO,CeO_2_ and Mn_2_O_3_ increased greatly and electric current produced under MW irradiation, whereas there are no phenomena without MW irradiation. The conductivity of semiconductor materials CuO,CeO_2_ and Mn_2_O_3_ could change with changing MW irradiation power (P_MW_), and the produced electric current could change with changing P_MW_ (Table S1-2). Although the produced electric current of TiO_2_ hasn’t changed with changing P_MW_ in a certain range, the conductivity of TiO_2_ has changed with changing P_MW_ (Table S2). However, for Al_2_O_3_, there are no changes, as show in Fig. 1. Similarly, Semiconductor TiO_2_,CuO,CeO_2_ and Mn_2_O_3_ were supported on activated carbon (AC) to prepare TiO_2_/AC, CuO/AC,CeO_2_/AC and Mn_2_O_3_/AC catalysts, when MW irradiates on these catalysts, we observe the same phenomenon. We found that the conductivity of TiO_2_/AC,CuO/AC,CeO_2_/AC and Mn_2_O_3_/AC catalysts suspension in the aqueous solution could change with changing P_MW_ (Table S3–5)_,_ and the produced electric current could change with changing P_MW_, as show in Fig. 2. These results indicated that MW irradiation excited semiconductor TiO_2_, CuO, CeO_2_, Mn_2_O_3_, TiO_2_/AC, CuO/AC, CeO_2_/AC and Mn_2_O_3_/AC catalysts to produce electric current. So that MW irradiation can excited semiconductor to generate electric current and electron-holes pairs (e^−^_cb_ + h^+^_vb_). Our experiments found that Microwave “photoelectric effect” exists under MW irradiation.

However, MW refers to the electromagnetic waves of 300 MHz–300 GHz, so the energy of MW photon is much lower than that of UV-light wave photon. Generally, MW photon could not excited metal or semiconductor such as TiO_2_ to generate electric current and electron-holes pairs. Our experiments observation in fact conform that Microwave “photoelectric effect” phenomenon exists when MW irradiation on semiconductor.

The above results also indicated that semiconductor TiO_2_,CuO,CeO_2_,Mn_2_O_3_ and SiC could be excited to generate electric current and electron-holes pairs (e^−^_cb_ + h^+^_vb_), whereas Al_2_O_3_ could not be excited to generate electric current and electron-holes pairs. So generation of Microwave “photoelectric effect” also depends on the dielectric properties of materials under MW irradiation. Generally, semiconductor materials have excellent dielectric properties and result in strong microwave absorption, for example, the relatively high loss tangent of CeO_2_,TiO_2_, and ZnO result in strong microwave adsorption^26^, low dielectric loss materials like Al_2_O_3_ almost do not interact with MW and do not absorb MW. Only when the energy of MW photon is higher than electric work function of materials, can the microwave “photoelectric effect” be produced.MW irradiation will lead to greatly reduce electric work function of semiconductor or MW catalysts.MW irradiation decreasing the electric work function of semiconductor catalysts depends on the properties of materials and its dielectric properties.

To reveal the nature/mechanism of Microwave “photoelectric effect” phenomenon, we give the explanation and elucidation for the principle of generation Microwave “photoelectric effect” under MW irradiation. The microwave is an electromagnetic wave and the corresponding irradiation is an electromagnetic wave irradiation. Based on the principle of quantum theory, photons of MW electromagnetic wave should/must interact on the microwave catalyst definitely under irradiation and thus there should be/existing the microwave “photoelectric effect”. However, photons of MW electromagnetic wave could not directly excite catalyst to produce electrons because generally the energy of MW photons (hν) is much lower than electric work function of materials such as MW catalyst TiO_2_/AC,CuO/AC and Mn_2_O_3_/AC (φ). In spite of the lower energy of MW photons, the influence of MW irradiation will lead to reduce electric work function of MW catalysts. MW irradiation decreasing the electric work function of MW catalysts depends on the properties of materials and its dielectric properties. MW irradiation can greatly decreased the electric work function of MW catalysts, only when hν > φ, could the microwave “photoelectric effect” be produced.

Based on the presented experiments, we consider that MW radiation can decrease the electronic work function of semiconductor from these views: On the one hand, owing to MW irradiation, MW energy could be absorbed by semiconductor rapidly depending on the dissipation factor of the semiconductor (loss tangent). Dissipation factor is the ratio of relative loss factor (ε″) to the permittivity (ε′). Permittivity is a relative measure of the MW energy density in the material and the relative loss factor is responsible for the internal loss mechanisms, such as the quantity of MW energy that is consumed in the semiconductor as heat energy. Therefore, a lossy material with a high relative loss factor can easily absorb MW energy^27^. Moreover, the quantum energy of MW could help to decrease the activation energy by making the vibration of molecules^28^. On the other hand, MW catalytic activity was mainly attributed to the microwave activation owing to dipolar polarization, conduction mechanism and interfacial polarization. Dipolar polarization contributing to most of MW heat energy results from intermolecular inertia. When the dipole is subjected to a high-frequency alternating electric field of the MW, rotation of the dipole can’t completely follow the rate of change of direction of the electric field. This leads to a time delay, causing abundant energy to be spent^29^.

Theoretically, any kind of electromagnetic wave is able to induce photoelectric effect^30^,as long as its frequency is higher than the limit frequency (it can be defined as the minimum frequency of a certain kind of material for the valence electron to be excited by electromagnetic wave to jump into the conduction band of the material.), and MW is not an exception. Interestingly, although the frequency (ν = 20 KHz–300 MHz) of ultrasonic wave is much smaller than the frequency (ν = 300 GHz–300 MHz) of microwave, ultrasonic wave can also excite to produce electron^31^. This indicates the microwave electromagnetic wave with a higher hν value is easier to excite to produce electron than ultrasonic wave.

Therefore, once catalyst with high relative loss factor and dielectric permittivity was subjected to MW irradiation, the electron hole pairs could be produced. This was probably attributed to the mechanisms of dipolar polarization, conduction mechanism and interfacial polarization. Dipolar polarization contributing to majority of MW heat energy results from intermolecular inertia. When the dipole is subjected to a high-frequency alternating electric field of the MW, rotation of the dipole can’t completely follow the rate of change of direction of the electric field, leading to a time delay and causing a substantial quantity of energy to be spent. MW can produce a lot of energy, leading to electrons in the high-energy state. Because electrons is instability in the high-energy state, which is in an active state and will produce transition. MW can produce a lot of energy, and high MW energy can directly effect on lowering the electric work function of semiconductor. Similarly, thermal energy or ultrasonic energy can also excite electrons. Accordingly, the electronic work function of the MW catalyst can be decreased by abundant thermal energy. Moreover, the quantum energy of MW can induce the vibration of molecules, which will help to decrease the activation energy of the reaction^32^.Consequently, semiconductor can be excited to generate electron-hole pairs under MW irradiation.

So the principle of generation microwave “photoelectric effect” could be revealed and elucidated as follow. On the one hand, the electric polarization effect of microwave electromagnetic field under MW irradiation lead to the dipole ordering result in conductivity rising, and then lowering the electric work function of semiconductor MW catalyst; MW irradiation could exist coupling effect of external electric field with internal electric field to lead to lowering the electric work function of semiconductor MW catalyst; On the other hand, MW energy partly transforms into materials interior energy level, and semiconductor or the whole MW catalyst such as TiO_2_/AC,CuO/AC,CeO_2_/AC and Mn_2_O_3_/AC could be heated to a higher temperature due to the effect of microwave thermal, producing a lot of thermal energy also lead to lower the electric work function of semiconductor MW catalysts. These two aspects effect above made the electric work function of semiconductor or MW catalyst to greatly decrease. So when the energy of photons are greater than electric work function of semiconductor or MW catalyst (hν > φ), MW catalyst could be excited directly to produce electrons under MW irradiation, and therefore generating electric current and electron-hole pairs (e^−^_cb_-h^+^_vb_) on the semiconductor or MW catalyst as like UV-light radiate on the metal to exist photoelectric effect. MW irradiation on semiconductor or the MW catalyst generating electric current and electron-hole pairs (e^−^_cb_-h^+^_vb_)could be called as MW “photoelectric effect”.

Interestingly, Huang^33^ studied the influence of electromagnetic fields on conductivity of aqueous NaCl solution at microwave frequency, and their results indicated that microwave energies were partly transformed to intermolecular energies of cluster, which made conductivity of aqueous NaCl solution interrelated with intensity of microwave. Their reported work^33^ also provides evidence on the change of conductivity under MW irradiation.

Based on the principle of our finding Microwave “photoelectric effect”, MW irradiation can directly excite semiconductor or MW catalyst to produce electron-holes pairs, the strong oxidizability of holes of generation electron-holes pairs under MW irradiation could be used for the oxidation reaction. Under MW irradiation, semiconductor catalyst such as Mn_2_O_3_/AC can be excited to produce electron-holes pairs, furthermore, the holes of generation electron-holes pairs on the semiconductor catalyst could react with H_2_O and OH to generate ∙OH for degradation of organics in wastewater.

**Figure 3:**
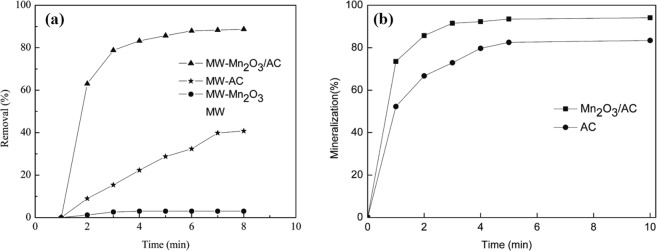
Effect of different catalysts on the degradation of 4-NP: (**a**) Removal; (**b**) Mineralization.

To further confirm to produce electron-holes pairs (e^−^_cb_ + h^+^_vb_) on the semiconductor catalyst by Microwave “photoelectric effect”, we use the principle of generation Microwave “photoelectric effect” to conduct the experiments for Microwave catalytic oxidation degradation of PNP in waste water using Mn_2_O_3_/AC as catalyst. In present of MW catalyst Mn_2_O_3_/AC, without adding oxidation agent such as H_2_O_2_, MW irradiation could catalytic oxidation degradation of organics in the wastewater and mineralize to CO_2_ and H_2_O (see Fig. 3). Without adding oxidation agent, where does the oxidation agent come from? MW irradiation can directly excite MW catalyst Mn_2_O_3_/AC to produce electron-holes pairs, and the holes with strong oxidation ability make H_2_O transform into ∙OH on the surface of the MW catalyst in the solution for degradation of organics. The results shown that Microwave “photoelectric effect” makes water adsorbing on the surface of Microwave catalyst to transform into ∙OH.

$$({{\rm{C}}}_{0}=100\,{\rm{mg}}/{\rm{L}};{{\rm{Q}}}_{{\rm{cat}}{\rm{.}}}=2\,{\rm{g}};{{\rm{P}}}_{{\rm{MW}}}=400\,{\rm{W}})$$

To well understand the generated ∙OH to oxidate degradation organics, adding ∙OH scavenging agent, the results shown that removal rate sharply decrease (Table S9). It is indicated that ∙OH is oxidation agent for oxidation degradation of organics. The fluorescence spectrums of the fluorescent substance produced by terephthalic acid reacting with ·OH in Fig. S7.aslo confirm the generation of ∙OH.

**Figure 4:**
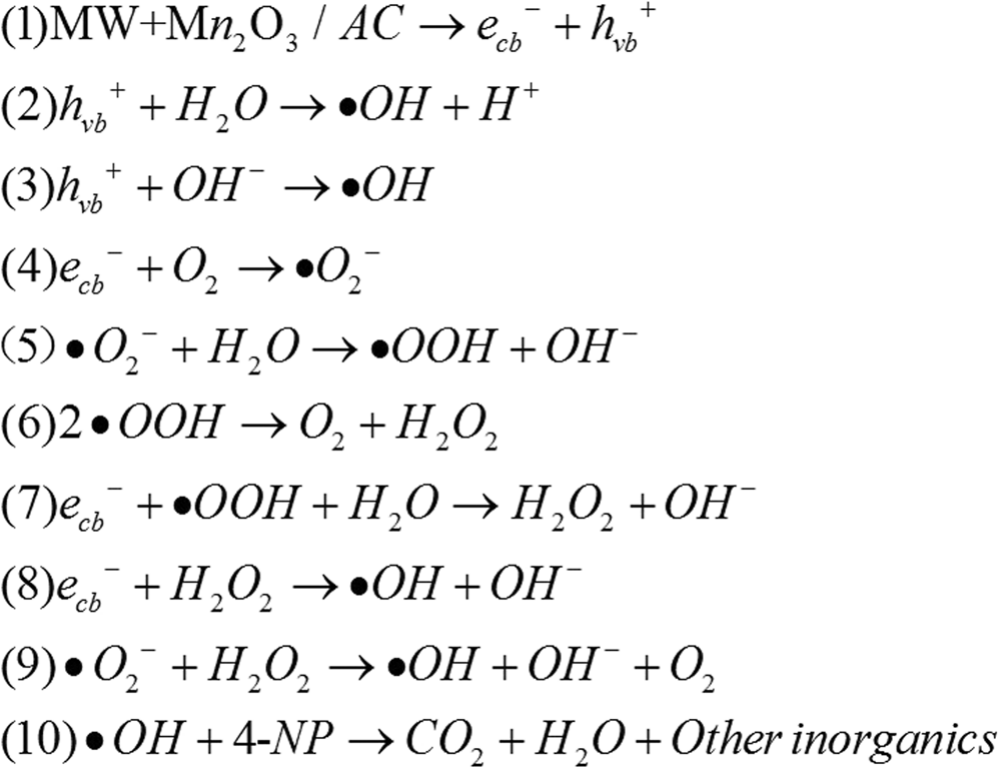
Illustration of generation of ∙OH in the process of MCOD.

According to the above results, ∙OH generated in the reaction process under MW irradiation is probably the main oxidant for the oxidation degradation of 4-NP. What is the mechanism for the generation of ∙OH under MW irradiation? Generally photons of MW electromagnetic wave could not directly excite catalyst to produce electron-hole pairs (e^−^_cb_-h^+^_vb_) because of the lower energy of photons. MW irradiation can greatly lower the electric work function of MW catalysts. So when the energy of photons are greater than electric work function of MW catalyst (hν > φ), MW catalyst could be excited directly to produce electron-hole pairs (e^−^_cb_-h^+^_vb_) under MW irradiation. The holes with strong oxidation ability react with the H_2_O molecules or OH^−^ on the surface of Mn_2_O_3_/AC particles, and electrons react with the O_2_ molecules dissolved in aqueous solution, respectively, producing the hydroxyl radicals (∙OH) and super oxygen radical anions (∙O_2_^−^). At last, unstable ∙O_2_^−^ also becomes ∙OH through series of chemical reactions. The generation process for ∙OH was shown in Fig. 4. Eqs (1–9). Owing to the strong oxidation ability, these ∙OH can oxidize 4-NP into CO_2_, H_2_O and other inorganic substances finally (Eq. (10)). Among them, Eqs (1–3) are the main process, and Eqs (4–9) are secondary, because the dissolved oxygen in reaction solution are limited. Obviously, as elucidation above, our finding MW “photoelectric effect” revealed clearly that mechanism of MW catalytic oxidation degradation of organics. Our previously work^34,35^ reported the MW catalytic oxidation degradation method (MCOD) for organic wastewater, and it is suggestion that hydroxyl radical (∙OH) generated under MW irradiation is the main oxidation agent. These results provide the evidence that hydroxyl radical (∙OH) generated by MW “photoelectric effect”. Quan *et al*.^36^ also observed that generation of hydroxyl radical in aqueous solution by microwave energy using activated carbon as catalyst also provides the evidence. Thus our finding MW “photoelectric effect” revealed the mechanism of hydroxyl radical (∙OH) generated in the MW catalytic oxidation degradation method.

Based on the principle of Microwave “photoelectric effect” above, MW irradiation can directly excite semiconductor or MW catalyst to produce electron-holes pairs, electron of generation electron-holes pairs could be used to make metal ion reduction into metal on the semiconductor support under MW irradiation. So our founding Microwave “photoelectric effect” can lead to develop new microwave reduction method for preparation of metal nanoparticles supported catalysts, and reveal the principle of the microwave reduction method. In spite of El-Shall M.S. and co-worker^25^ have reported Microwave synthesis of supported Au and Pd nanoparticle catalysts. However, the mechanism of formation was not explored in detail^26^. Narayanan^26^ presented detailed and new insights on the formation of nanoparticle hybrids containing metal nanoparticles on oxide supports using the microwave reduction method. They observe that as expected on MgO and TiO_2_ substrates, uniform decoration of Au nanoparticle is observed whereas very few particles are present on Al_2_O_3_ and SiO_2_. So their results just in time also provide evidence for our findings. Only oxide support is suitable semiconductor materials, Microwave “photoelectric effect” could made metal ion reduction into metal nanoparticles supported on the oxide support.

Further measurement experiments for value of electric work function of materials under MW irradiation could be carried out and suitable materials as MW catalysts should be investigated.

## Conclusion

In summary, we report an unexpected, Microwave “photoelectric effect”, when MW irradiates on the semiconductor materials, leading to generate electric current and electron-holes pairs (e^−^_cb_ + h^+^_vb_), on the semiconductor materials and on the MW catalyst. We found that microwave “photoelectric effect” exists when MW irradiates on the semiconductors and microwave “photoelectric effect” made water on the surface of Microwave catalyst to transform into hydroxyl radical (∙OH). This study has revealed the principle of generation Microwave “photoelectric effect” under MW irradiation, and the mechanism of MW reduction method for preparation of nano-particle metal supported catalysts. Our findings challenge the classic view of MW irradiation only as heating method, which cannot excite to produce electric current and electron-holes pairs (e^−^_cb_ + h^+^_vb_). Our findings will open new field to use MW technology for MW catalytic oxidation degradation of organic in the wastewater, and for MW reduction method of metal supported catalysts preparation.

## Experimental Section

### Preparation of MeO_x_ nanoparticle and MeO_x_/AC catalysts

MeO_x_(Me = Cu, Ce, Mn, Ti, Al) nanoparticle were prepared with a coprecipitation method.10 g of Cu(NO_3_)_2_·3H_2_O, Ce(NO_3_)_3_·6H_2_O, 50%Mn(NO_3_)_2_ Solution, Ti(SO_4_)_2_, Al(NO_3_)_3_·9H_2_O was dissolved in ethanol, respectively, and then 1 ml polyethylene glycol was added dropwise to the above mixture under continuous vigorous stirring. The resulting solution was adjusted the pH to 10 by the addition of NaOH and then irradiated under microwave (the working power of 214W) in a pulsated way for 10 min. After filtration and being washed with deionized water and ethanol,the solid obtained was dried at 80 °C for 12 hours and calcination at 500 °C for 3 hours.

MeO_x_/AC were prepared with an impregnation method. Activated carbon (designated as AC, Φ3.0 mm, SinopharmChemicalReagent Co., Ltd.) was pretreated with the boiling deionized water, and then dried at 80 °C for 12 h. The desired amount of AC was impregnated with aqueous solution of Mn(NO)_2_, Ce(NO_3_)_3_, Ti(SO_4_)_2_, Cu(NO_3_)_2_, respectively, at room temperature for 12 h followed by drying at 80 °C for 12 h. The MeO_x_/AC samples were obtained after calcining at 250 °C for 2 h. The characterization results of MeO_x_/AC were listed in Figs S3–S6 and Table S7.

### Experimental method

Microwave single-mode experiments was carried out in a HY-SG1500 microwave tubular furnace (Hunan Hua’e Microwave Technology Co. Ltd), as shown in Fig. S1. A certain amount of MeO_x_ powder was placed in a silica crucible and pressed with silica wool, and then, the silica crucible or a carborundum plate, connected with two wire at the bottom both sides, was irradiated under various MW power levels for a certain time. The wire was winded with aluminum foil to eliminating microwave interference. The resistance and current of the MeO_x_ was measured by a Fluke AVOmeter.

Microwave multi-mode experiments was carried out in a COOLPEX-E Microwave apparatus (Shanghai Yiyao technology) of 2450 MHz frequency, as shown in Fig. S2. 0.5 g of MeO_x_/AC or AC was added to 100 mL of deionized water, and then, the suspension was irradiated under various MW power levels for a certain time under continuous magnetic stirring. The resistance and current of the suspension was measured by a Fluke AVO meter which connected with two wires from the bottom of 3 mouth flask. The wire was also winded with aluminum foil to eliminating microwave interference.

The experiments for Microwave catalytic oxidation degradation of PNP in waste water using Mn_2_O_3_/AC as MW catalyst was conducted in detail seeing supporting information section II.

## Supplementary information

Supplementary Information
