# Supplementary material for: Microwave irradiation directly excites semiconductor catalyst to produce electric current or electron-holes pairs
Source: Sci Rep. 2019 Apr 2;9:5470. doi: 10.1038/s41598-019-41002-w (PMC6445116; doi:10.1038/s41598-019-41002-w)
Supplement: Supplementary file 1 — Supplementary Information [file 41598_2019_41002_MOESM1_ESM.docx]

Supporting information

**Microwave irradiation directly excites semiconductor catalyst to produce electric current or electron-holes pairs**

Jicheng Zhou ^*^, Zhimin You, WentaoXu,ZhimingSu, YinQiu,Lingfei Gao, Cheng Yin, LixinLan

Key Laboratory of Green Catalysis and Chemical Reaction Engineering of Hunan Province, School of Chemical Engineering, Xiangtan University, Xiangtan 411105, Hunan Province, PR China

**Section I MW irradiation on semiconductor experiments**

**S1.Experimental**

**S1.1 *Preparation of materials***

**S1.1.2Preparation of MeO_x_nanoparticle**

MeO_x_(Me= Cu,Ce,Mn, Ti, Al) nanoparticle were prepared with a coprecipitation method. 10 g ofCu(NO_3_)_2_·3H_2_O, Ce(NO_3_)_3_·6H_2_O, 50%Mn(NO_3_)_2_ Solution, Ti(SO_4_)_2_, Al(NO_3_)_3_·9H_2_O was dissolved in ethanol, respectively, and then 1ml polyethylene glycol was added dropwise to the above mixture under continuous vigorous stirring. The resulting solution was adjusted the pH to 10 by the addition of NaOH and then irradiated under microwave(the working power of 214W) in a pulsated way for 10 min. After filtration and being washed with deionized water and ethanol, the solid obtained was dried at 80 ℃ for 12 hours and calcination at 500℃ for 3 hours.

**S1.1.2Preparation of MeO_x_/AC**

MeO_x_/AC were preparedwith an impregnation method.Activated carbon (designated as AC, Φ3.0mm, SinopharmChemicalReagent Co., Ltd.) was pretreated with the boiling deionized water, and then dried at 80 °C for 12 h. The desired amount of AC wa simpregnated with aqueous solution of Mn(NO)_2_, Ce(NO_3_)_3_, Ti(SO_4_)_2_ , Cu(NO_3_)_2_, respectively，at room temperature for 12 h followed by progressivedrying at 80 °C for 12 h. The MeO_x_/AC samples were obtained after calcining at 250 °C for 2 h.

***S1.2 Experimentalapparatus***

Microwave single-mode [experiment](app:ds:experiment)s were carried out in a HY-SG1500 microwave tubular furnace (Hunan Hua'e Microwave Technology CO., LTD). Microwave irradiates directly to the sample pool where a silica crucible filled with [sample to be tested](file://C:\Documents%20and%20Settings\yzm\Local%20Settings\Application%20Data\youdao\Dict\Application\5.0.35.9705\resultui\app:ds:sample%20to%20be%20tested) was placed. The reflected or transmitted microwave was absorbed by the silicon carbide plates covered at the top of the sample pool. The experimental apparatusis shown in Fig.S1.


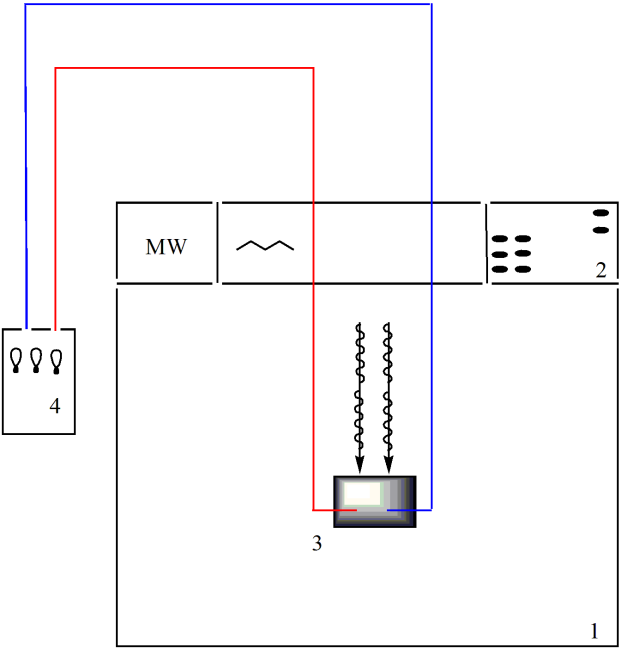


**Fig. S1** Microwave single-mode Experimental apparatus

1 - MW generator, 2 - parameter adjusting button, 3- sample pool ,4-Fluke AVOmeter

Microwave multi-mode [experiment](app:ds:experiment)s were carried out in a COOLPEX-E microwave apparatus (Shanghai Yiyao technology) of 2450 MHz frequency, the experimental apparatus is shown in Fig.S2.


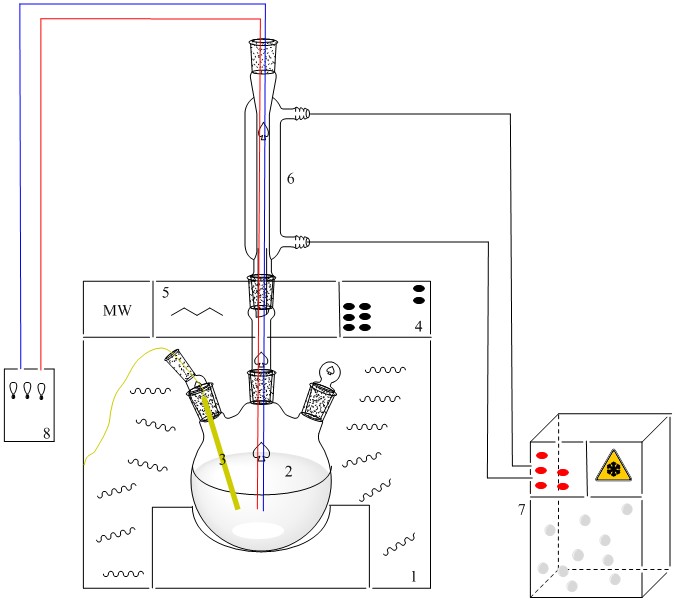


**Fig. S2** Microwave multi-mode experimental apparatus

1 - MW generator, 2 – 3 mouth flask, 3- thermocouple, 4 - parameter adjusting button,5-screen, 6- condenser pipe, 7 - circulation condensate pump, 8-Fluke AVO meter

***S1.3 Experimental method***

**S1.3.1 Microwave single-mode** [**experiment**](app:ds:experiment)**s**

A certain amount of MeO_x_ powder was placed in a silica crucible and pressed with [silica](app:ds:silica)[wool](app:ds:wool). And then, the silica crucible or a carborundum plate, connected with two wire at the bottom both sides, was irradiated under various MW power levels for a certain time.The wire was winded with aluminum foil to eliminating microwave interference. The resistence and current of the MeO_x_ was measured by a Fluke 15B AVO meter.

**S1.3.2 Microwave multi-mode** [**experiment**](app:ds:experiment)**s**

0.5 g of MeO_x_/AC or AC was added to 100 ml of deionized water, and then, the suspension was irradiated under various MW power levels for a certain time under continuous magnetic stirring. The resistance and current of the suspension was measured by a Fluke 15B AVOmeter which connected with two wires from the bottom of 3 mouth flask. The wire was also winded with aluminum foil to eliminating microwave interference.

**S2. Results and discussion**

**Fig.S3.** XRD patterns of MeO_x_/AC

TableS1.The effect of P_MW_ on produced electric currentoverMeO_x_

| MW irradiation power (W) | Current(mA) | | | | |
| --- | --- | --- | --- | --- | --- |
|  | CeO_2_ | Al_2_O_3_ | MnO_2_ | CuO | TiO_2_ |
| 0 |  |  | 0 | 0 | 0 |
| 50 | 0.006 |  | 0 | 0 | 0 |
| 100 | 0.028 |  | 0.03 | 0 | 0 |
| 150 | 0.054 |  | 0.08 | 0.182 | 0 |
| 200 | 0.047 | 0.002 | 0.14 | 0.261 | 0 |
| 250 | 0.043 | 0.002 | 0.24 | 0.213 | 0 |
| 300 | 0.067 | 0.003 | 0.205 | 0.023 | 0 |
| 350 | 0.035 | 0.002 | 0.433 | 0.31 | 0 |
| 400 | 0.094 |  | 0.051 | 0.469 | 0 |
| 500 | 1.659 |  | 0.033 | 0.411 | 0 |

TableS2.The change of Alternating current resistance of MeO_x_ with P_MW_

| MW irradiation power(W) | Alternating current resistance (MΏ） | | | Alternating current resistance（kΏ） | | |
| --- | --- | --- | --- | --- | --- | --- |
|  | CeO_2_ | TiO_2_ | Al_2_O_3_ | MnO_2_ | CuO | SiC |
| 0 |  |  | 1.1 | 5.69 |  | 7.5 |
| 50 |  | 15.46 | 1.1 | 4.71 |  |  |
| 100 |  | 14.83 | 1.1 | 1.67 | 12.5 |  |
| 150 | 10.01 | 14.01 | 1.1 | 0.722 | 11.5 |  |
| 200 | 5.45 | 11.33 | 1.1 | 0.15 | 1.229 | 2.4 |
| 250 | 3.924 | 8.87 | 1.1 | 0.004 | 0.084 |  |
| 300 | 2.458 | 7.29 | 1.1 | 0 | 0.0257 | 2.5 |
| 350 | 0.345 | 5.85 | 1.1 | 0 | 0 | 2.5 |
| 400 | 0 | 4.88 | 1.1 | 0 | 0 | 1.2 |
| 450 | 0 | 4.28 | 1.1 | 0 | 0 | 1 |
| 500 | 0 | 3.98 | 1.1 | 0 | 0 | 0.89 |
| 600 |  |  |  | 0 | 0 | 0.5 |

TableS3.The change of temperature of MeO_x_/ACwith P_MW_

| MW irriadition power(W) | Temperature(℃) | | | | |
| --- | --- | --- | --- | --- | --- |
|  | CeO_2_/AC | TiO_2_/AC | CuO/AC | Mn_2_O_3_/AC | AC |
| 0 | 13 | 12 | 13 | 12 | 12 |
| 100 | 13 | 12 | 13 | 12 | 12 |
| 200 | 69 | 58 | 61 | 29 | 56 |
| 300 | 92 | 96 | 81 | 47 | 98 |
| 400 | 136 | 120 | 38 | 79 | 120 |
| 500 | 148 |  | 120 |  | 129 |
| 600 | 152 |  | 123 |  | 147 |

TableS4.The effect of P_MW_ on produced electric current over MeO_x_/AC

| MW irradiation power(W) | Current（mA） | | | | |
| --- | --- | --- | --- | --- | --- |
|  | CeO_2_/AC | TiO_2_/AC | CuO/AC | Mn_2_O_3_/AC | AC |
| 200 | 0.091 | 0.348 | 0.268 | 0.087 | 0.239 |
| 300 | 0.112 | 0.348 | 0.275 | 0.155 | 0.278 |
| 400 | 0.159 | 0.402 | 0.37 | 0.161 | 0.302 |
| 500 | 0.168 | 0.548 | 0.268 | 0.238 | 0.309 |
| 600 | 0.265 | 0.532 | 0.285 | 0.203 | 0.372 |

TableS5.The change of Alternating current resistance of MeO_x_/ACwith MW irradiation power P_MW_

| MW irradiation power(W) | Alternating current resistance（kΏ） | | |
| --- | --- | --- | --- |
|  | CuO/AC | CeO_2_/AC | AC |
| 200 | 97.5 | 97.4 | 50.1 |
| 300 | 62.9 | 36.2 | 35.8 |
| 400 | 27.9 | 11.8 | 10.8 |
| 500 | 11.7 | 4.7 | 2.6 |

We have performed comparative experiments that using conventional oil bath heating method to heat the solution containing the semiconductor catalyst to 100 °C, and found it cann’t produce current and the resistances do not decline. It indicates that the semiconductor material absorbs the microwave which can cause the "thermal effect" and the change of internal energy level. This can result tothe electric work function of the semiconductor material become very small.So thatit can be observed the microwave "photoelectric effect" in experiments.

Table S6.Effect of treatment process on produced electric current over MeOx/AC(P_MW_ = 300W, the Temperatue of Traditional heating = 100 ^o^C)

| Treatment process | Current（mA） | | | | |
| --- | --- | --- | --- | --- | --- |
|  | CeO_2_/AC | TiO_2_/AC | CuO/AC | Mn_2_O_3_/AC | AC |
| Traditional heating | 0 | 0 | 0 | 0 | 0 |
| Microwaveirradiation | 0.112 | 0.348 | 0.275 | 0.155 | 0.278 |

**Section II Microwave catalytic oxidation degradation of PNP and phenol in waste water using Mn_2_O_3_/AC as catalyst**

**S3. Experimental**

***S3.1 Preparation of Mn_2_O_3_/AC***

The novel microwave catalyst of activated carbon supported Mn_2_O_3_ was prepared using impregnation method. Activated carbon were seethed by distilled water for 2h at first and rinsed repeatedly then the washed [activated](app:ds:activated) [carbon](app:ds:carbon) was dried at 110℃ for 12h in oven. Pretreated [activated](app:ds:activated) [carbon](app:ds:carbon) were impregnated with aqueous solution of manganese nitrate for 12 h at room temperature and dried for 12h atrelatively temperatures of 65 ℃. Finally the dried samples were calcined at 250℃ for 2h in muffle oven.The microwave catalyst used in the experiment is 3wt % Mn_2_O_3_/AC.

***S3.2 Characterization of*** ***Mn_2_O_3_/AC***

An X-ray diffraction (XRD) analysis was performed on the Rigaku D/MAX-2550 diffractometer using Cu Ka radiation (λ=0.1542 nm) and graphite monochromator. The specific surface area and pore structure of the supported Mn_2_O_3_/AC was characterized by N_2_ adsorb-desorbs analysis with micrometritics NOVA-2200e at -77℃.Fouriertransform infrared (FT-IR)spectra analysis was carried out using FT-IR spectrometer(Spectrom One, PE Company, USA) to investigate the presence of Mn_2_O_3_/AC.The surface morphology of the catalyst was observed by scanning electron microscopy (SEM).

***S3.3Degradation of 4-nitrophenol***

The microwave catalytic oxidation degradation of 4-NP was carried out in a microwave generator (CEM Mars-5, USA) which could control microwave power，reaction time and temperature. 100ml of aqueous 4-NP solution was placed in a three-neck flask connected with a refluxing device. A fit amount of catalyst was suspended in the solution. The catalyst dosage, microwave power, reaction time and initial concentration are within the range of 0-2g, 0-800 W, 0-10 min and 0-1000 mg/L respectively. The temperature of reaction solution rising to 100℃ in about 2 min and then remained unchanged under microwave irradiation. After reaction, it was cooled to a certain temperature in 8 min before reaction solution analysis. The initial and processed concentration of 4-NP solution was analysisby a high-performance liquid chromatograph (HPLC) (Perkin Elmer, Series 200，America) equip with UV detector at 326 nm detection wavelength. The TOC of the reaction solution was determined using TOC analyzer (TOC-L CPH CN200, Shimadzu).

**S4. Results and discussion**


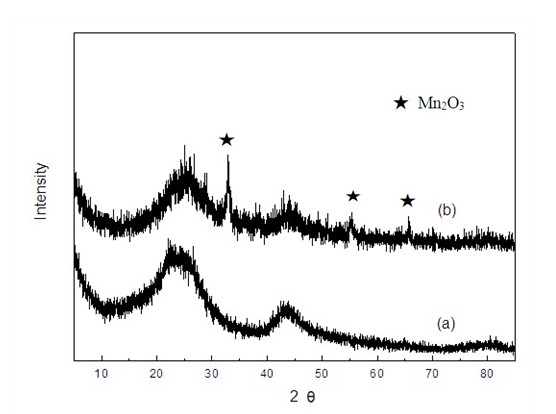


**Fig.S4.** XRD patterns of Mn_2_O_3_/AC catalysts

Table S7. Testing results of N_2_ adsorb-desorbs

| sample | S_BET_/m^2^.g^-1^ | d/nm | V/cm^3^. g^-1^ |
| --- | --- | --- | --- |
| AC | 801.079 | 1.171 | 0.167 |
| Mn_2_O_3_/AC | 754.547 | 1.199 | 0.097 |

**Fig.S5.** FT-IR spectra of catalysts:(a) AC; (b) Mn_2_O_3_/AC


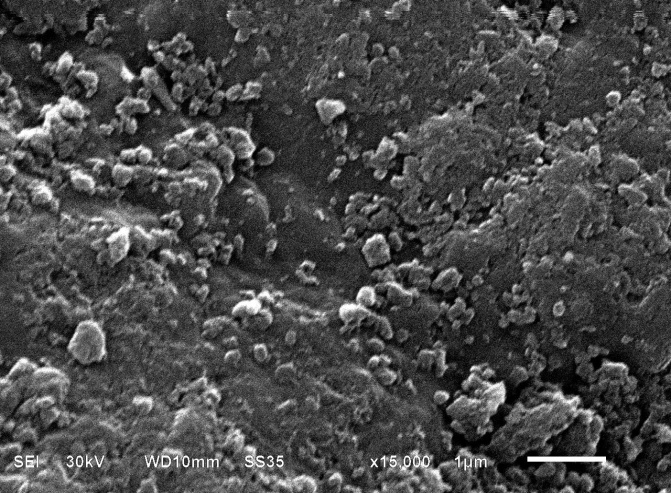


**Fig.S6.**SEM spectra of Mn_2_O_3_/ACcatalysts

Table S8. Effect of treatment process on removal of 4-NP (P_MW_ = 400W, C_0_ = 100 mg/L, t = 5 min, Q_cat._ = 2 g, T = 100 ^o^C)

| Treatment process | Removal of PNP (%) | |
| --- | --- | --- |
|  | AC | Mn_2_O_3_/AC |
| Adsorption | 9.76 | 8.24 |
| Traditional heating | 18.28 | 17.49 |
| Microwave catalysis | 87.93 | 99.56 |

TableS9.Effect of adding radical scavengers on the removal of 4-NP

| Radical scavenger | Removal/% |
| --- | --- |
| None | 99.6 |
| thiourea | 91.2 |
| dimethylsulfoxide | 88.5 |
| L-histidine | 86.4 |
| Vitamin C | 72.3 |

(C_0_=100 mg/L, Q cat.=2 g, P_MW_=400 W and T=5min)

We have also carried out the comparative experiments through adding O_2_ during the reaction. There is no substantial change after adding O_2_ over Mn_2_O_3_/AC under microwave irradiation, indicating that H_2_O in aqueous solution are enough to generate 
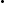
OH and extra O_2_ is not necessary.


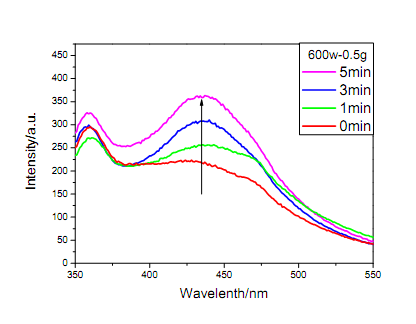


**Fig.S7.**The fluorescence spectrums of the fluorescent substance produced byterephthalicacid reacting with ·OH. (Catalyst dosage = 0.5 g, MW power = 600 w.)

Table S10. Dielectric properties of the various oxide Supports [S1]

| material | Dielectric constant | Loss tangent |
| --- | --- | --- |
| Al_2_O_3_ | 11.6 | 0.00005 |
| Al_2_O_3_ | 9.4 | 0.00007 |
| SiO_2_ | 3.8 | 0.00016 |
| MgO | 9 | 0.007 |
| TiO2 | 50 | 0.002 |
| CeO2 | 23 | 0.001 |
| ZnO | 8.3 | 0.2 |

**S5.The mechanism of microwave catalytic oxidation degradation of organics in waste water using MeO_x_/AC as catalyst.**

In present of MW catalyst MeO_x_/AC, MW irradiation made organics removal in the wastewater and oxidation degradation of organics and mineralize to CO_2_ and H_2_O. MW irradiation can directly excite MW catalyst MeO_x_/AC to produce electron-holes pairs, and the holes with strong oxidation ability make H_2_O transform into •OH on the surface of the MW catalyst in the solution for degradation of organics. The results shown that Microwave “photoelectric effect” make water adsorbing on the surface of Microwave catalyst to transform into hydroxyl radical(•OH).

$$\begin{matrix} (1)\text{MW+M}e\text{O}_{x}/AC\to e_{cb}^{-}+h_{vb}^{+} \\ (2)h_{vb}^{+}+H_{2}O\to\bullet OH+H^{+} \\ (3)h_{vb}^{+}+OH^{-}\to\bullet OH \\ (4)e_{cb}^{-}+O_{2}\to\bullet O_{2}^{-} \\ \text{（}5\text{）}\bullet O_{2}^{-}+H_{2}O\to\bullet OOH+OH^{-} \\ (6)2\bullet OOH\to O_{2}+H_{2}O_{2} \\ (7)e_{cb}^{-}+\bullet OOH+H_{2}O\to H_{2}O_{2}+OH^{-} \\ (8)e_{cb}^{-}+H_{2}O_{2}\to\bullet OH+OH^{-} \\ (9)\bullet O_{2}^{-}+H_{2}O_{2}\to\bullet OH+OH^{-}+O_{2} \\ (10)\bullet OH+organics\to CO_{2}+H_{2}O+Other inorganics \end{matrix}$$

Scheme 1. Illustration of generation of •OH in the process of MCOD

The process of microwave catalytic degradation of organic compounds can be show in Fig. S8.

**MW electromagnetic wave irradiation**

Fig.S8. Reaction pathway of organics in MCROD process

**Reference**

[S1]Erumpukuthicka, A. A., Paromita, K., Parag, A. D., Giridhar, M. & Narayanan, R. New insight into selective heterogeneous nucleation of metal nanoparticles on oxides by microwave-assisted reduction: rapid synthesis of high-activity supported catalysts, ACS Nano, 5, 8049-8061 (2011).
